# Navigating the Tower of Babel: The Epistemological Shift of Bioinspired Innovation

**DOI:** 10.3390/biomimetics5040060

**Published:** 2020-11-09

**Authors:** Taryn Mead, David Coley, D. Scott Borden

**Affiliations:** 1 School of Business, Western Colorado University, Gunnison, CO 81231, USA; sborden@western.edu; 2 Department of Architecture & Civil Engineering, University of Bath, Bath BA2 7AY, UK; dac33@bath.ac.uk

**Keywords:** bioinspired innovation, biomimicry, biomimetics, bioinspired design, epistemology, sustainability

## Abstract

The disparity between disciplinary approaches to bioinspired innovation has created a cultural divide that is stifling to the overall advancement of the approach for sustainable societies. This paper aims to advance the effectiveness of bioinspired innovation processes for positive benefits through interdisciplinary communication by exploring the epistemological assumptions in various fields that contribute to the discipline. We propose that there is a shift in epistemological assumptions within bioinspired innovation processes at the points where biological models derived from reductionist approaches are interpreted as socially-constructed design principles, which are then realized in practical settings wrought with complexity and multiplicity. This epistemological shift from one position to another frequently leaves practitioners with erroneous assumptions due to a naturalistic fallacy. Drawing on examples in biology, we provide three recommendations to improve the clarity of the dialogue amongst interdisciplinary teams. (1) The deliberate articulation of epistemological perspectives amongst team members. (2) The application of a gradient orientation towards sustainability instead of a dichotomous orientation. (3) Ongoing dialogue and further research to develop novel epistemological approaches towards the topic. Adopting these recommendations could further advance the effectiveness of bioinspired innovation processes to positively impact social and ecological systems.

## 1. Introduction

The translation of biological metaphors and analogies into design, organizational, and manufacturing solutions is frequently viewed as an expansive and provocative approach to problem solving [1,2,3]. Various terms such as bioinspired innovation (BII), biomimicry, biomimetics, bionics, etc., have been used to label this approach to problem solving, with each label having its devotees. For most technically- or innovation-driven disciplines, the subject of epistemology and theories of knowledge are rarely a source of conversation and debate outside of limited circles (see Appendix A for a glossary of terms related to this topic.) A practical focus on the material aspects of innovation outcomes does not typically instigate a discourse of ways of knowing as evoked by epistemological inquiry. If it did, it would involve questions such as “What impact will this innovation decision have on human perceptions of ‘nature’? How will it influence indigenous people’s relationship to the land? What impact will this decision have on the inherent rights of nature to flourish?” However, in more philosophical circles of discourse, the underlying assumptions about normativity, ethics, and epistemological positions are regular topics [4,5]. The disparity between disciplinary approaches has created a cultural divide that is stifling to the overall advancement of BII as a tool for sustainable societies.

Epistemological underpinnings—whether acknowledged or not—shape the normative aspects of innovation intentions and outcomes that vary substantially across disciplines and their associated subcultures. “Epistemology is defined as the study of how we know what we know. These lines of inquiry explore how we perceive the world around us and how those perceptions influence our interpretations of our own knowledge and reality [6].” For most technically- and innovation-driven researchers, epistemological and ontological pretenses are not systematically considered when conceptualizing our experiences in everyday life. Nevertheless, what we know and how we come to know it are influential for the ways that we communicate our research methods and outcomes to various audiences. For example, a biologist pursuing biological metaphors and analogies may be inclined to communicate the technological and efficiency benefits of their research findings, whereas a social scientist may communicate the same innovation but focus more on the social and health benefits for the wider society. This is not to assign a value judgment on either approach, only to say that differences exist in the valuation of biological metaphor- and analogy-based outcomes, and possibly some differences are encapsulated in the distinctive label (bioinspired innovation, biomimicry, biomimetics, bionics, etc.) used.

Epistemology also influences how we articulate the various values that our work creates for our workplaces and communities. Basic research may seem a benign activity, but the pursuit of knowledge is not a socially or ecologically neutral endeavor [7]. Common epistemological positions for subjects relevant to BII (i.e., sustainability, transdisciplinarity, biology, design, and engineering) are discussed in the following sections. We demonstrate how these positions differ across disciplines and how this variation influences perceptions of sustainability outcomes. This paper introduces the concept of an *epistemological shift:* a three-phase transition of epistemological positions embedded in a BII process that takes the user from a reductionist (position #1) derived biological observation towards a socially-constructed (position #2) interpretation of the biological model in the abstraction of design principle, which is ultimately translated into a culturally relevant application wrought with the complexity and multiplicity of a more realist epistemology (position #3). For the purposes of explanation, this paper provides a simplified three-phase model to demonstrate the concept, but it is likely that various users move through this epistemological shift in numerous iterations and perhaps in varied sequences throughout a design or innovation process.

This epistemological shift is integral to BII and yet is not integrated into practical considerations for sustainability-oriented outcomes. While a biological model may be sustainable, one should not assume that any translated application of a biological model is also sustainable. For instance, while the first reductionist phase of a BII process would reveal the growth and materials of a lotus leaf as a benign component of an aquatic ecosystem, one should not assume that a surface designed to perform similar to a lotus leaf using nanoparticles (a socially constructed useful application of the design principle) would also be a benign component of its ecosystem. The lotus effect design principle may result in some characteristics that are more ecologically friendly compared to existing products on the market; however, it is debatable whether this is a sustainable innovation when considered in the complex context of the entire product life cycle (exemplifying the realist position of multiplicity and complexity). As we attempt to exemplify below, different practitioners and theorists frame their initial thoughts and language regarding biological phenomena in different ways, and this most likely influences the transition of epistemological positions embedded in a BII process in the path from a reductionist observation to socially-constructed interpretation to application.

Many bioinspired innovators are in the challenging position of defining what sustainability means in the context of their work. Innovators—including biologists, designers, engineers, and others—apply a broad range of disciplinary perspectives to qualify their decision-making for sustainability in BII [8]. Due to differing and commonly overtly embedded epistemologies within each discipline, communicating intentionally and avoiding metaphors may be difficult. The following sections explore the epistemologies of various disciplines that influence how BII and sustainability are conceptualized and implemented. Special attention is given to the shortcomings of these epistemologies within the needs of bioinspired innovation to avoid naturalistic fallacy.

## 2. Epistemologies of Biology

A requisite step in BII is the inclusion of biological strategies and principles [9]. This biological knowledge is derived from a rigorous process of the hypothesis-driven scientific method to conduct research. Derived from a historical context, a purely positivist epistemology and related methodologies view reality as an obvious and knowable phenomenon that can be understood through observation and validated by measurement [10]. This logical positivism is based on four key assumptions: (1) “methods of understanding reality are independent of culture”; (2) “reality is independent of methods of understanding”; (3) “reality can be understood in terms of universal laws”; and (4) “reality can be understood through one set of universal laws” ([11], pp. 43–44). Hypothetically speaking, the complete understanding of the universe could be enabled through an extensive checklist of knowledge acquired through a process of hypothesis testing. Every scientific finding represents another tick on the checklist of truths and fallacies about the operating of the universe. A positivist approach requires reproducibility as a key criterion, allowing for others to come to identical conclusions using an identical experimental model [12]. Given that biological knowledge is a foundational component of BII, the adherence to a positivist epistemology and reductionist logic is a critical aspect of legitimization in research and innovation.

However, some critics argue that while a well-designed study includes controls to temper our tendency towards confirmation bias, neutrality in science is an unobtainable, idealized state [13]. Kuhnian assertions that we are simultaneously guided by the matter that we are studying and the paradigm of science from which we view it are increasingly accepted in numerous disciplines [14]. An unexamined positivist approach can produce a level of reductionism that simplifies the complexity of reality, inadvertently minimizes the influence of multifactorality, and ineffectively isolates variables that are influenced by human agents and perceptions [15]. This simplification can lead to decontextualized knowledge that lacks relevance in timely societal issues, particularly in the case of sustainability research that is frequently positioned in a setting of socioecological systems [12]. For example, a rise in atmospheric carbon and increased temperatures associated with climate change are not problematic for humanity unless contextualized as a source of societal unrest, a detriment to human health, and disruption to the world economy.

While the use of positivist lines of inquiry is a critical aspect of BII, they concurrently do not address questions of normativity for sustainability or issues of ethics and cultural appropriateness of technology. Questions related to societal impacts and value-laden decisions that drive innovation processes are methodologically excluded from the problem-solving process. Without interpretivist and constructivist perspectives in the creation of BII-based knowledge and innovation, research questions are focused on how to create novel innovations and whether they are “Biomimetic or not?” rather than “Who does this biomimetic innovation serve and to what ends?”

## 3. Epistemologies of Design and Engineering

The design and engineering components of BII are—contrary to reductionist approaches—largely guided by the interpretation of biological phenomena into socially relevant contexts and constructed solutions. As Alexandra Daisy Ginsberg articulates, “The designer is better equipped as a generalist, in contrast to the scientist, who is a specialist, an expert in the detail of how things work, not whom they work for” [16]. It is largely accepted that design has no established epistemic foundation or even “epistemological tendencies” in design processes [17] and relatedly, discussions of the epistemological foundations of engineering are varied and context-specific [18,19,20].

Relatedly, an engineer’s skill set could be considered as a combination of the qualities of a scientist, a sociologist, a designer, and a doer. The engineer relies on reductionism when applying the basic sciences for the technical aspects of their work. Concurrently, they apply interpretivist and constructivist epistemologies when problem-solving with the skills of a sociologist, designer, and doer [19].

Constructivism and interpretivism are epistemological positions, which assert that human perceptions (mediated through language) create knowledge that is inseparable from human and social constructions. This position argues that while the universe is independent of the human mind, it cannot be understood without the lenses of individual and social constructions [21]. In the context of BII, this implies that a design and/or engineering innovation process is a socially constructed translation of a biological phenomenon that cannot be separated from the agency, intentions, and perceptions of the interpreter. The resulting innovation is a reflection of the innovator, rather than a reflection of the biological phenomenon, per se. Whether intentional or not, this translational aspect of BII is indeed a reflection of societal norms, cultural standards, and research priorities. However, the results of some BIIs, which may be a close emulation of a biological phenomenon, remain irrelevant in a social context, with prototypes doomed to a life of desertion on a research lab shelf or to be memorialized in a white paper.

## 4. Reconciling the Epistemological Shift of BII

**Figure 1:**
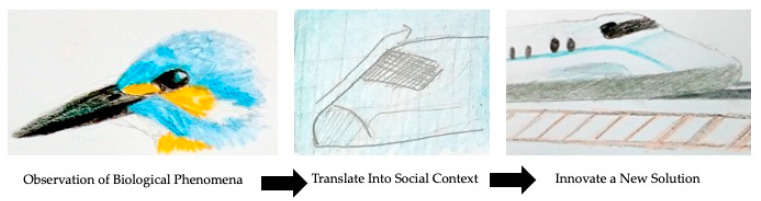
Basic bioinspired innovation process.

BII has been characterized as having three basic steps: (1) observation of biological phenomena (described here as generally a positivist approach), (2) translation of phenomena into a design principle and social context (described here as a constructivist or interpretivist approach) and (3) creation of new innovation based on the design principle [9]. Figure 1 demonstrates this process.

This final step could be viewed from a realist epistemology, which states that the physical world exists independently of our perceptions of it, and concurrently, our sensory perceptions of it reflect how it actually is. Critical realists share with positivists a value of the objective world, its patterns, and related generalizations. However, similarly to constructivists, realism critiques positivism as being too shallow in its limitations to observable phenomena and suggests that the unobservable mechanisms that produce a phenomenon are undervalued. Critical realists are interested in the theoretical and observable complexities that underlie social phenomena [22]. The realist perspective reflects some epistemological multiplicity that embraces complexity and irreducibility as inevitable.

For the innovator that merges a reductionist observation with a constructed social context, the resulting innovation is open for a multitude of interpretations from users, scholars, and scientists. Each of these interpretations is reflective of the unique perspective of the observer to evaluate the relevance and efficacy of the innovation within their own complex realities. The very process of BII takes the user seamlessly across epistemological boundaries, without addressing the existence of these boundaries whatsoever.

## 5. Sustainability and BII

Effective communication between disciplines is particularly relevant when applying BII for sustainability-oriented innovations [8,23,24,25]. Sustainability science, as a practice, encompasses multiple scales, various dynamics, diverse actors, and systemic perspectives. It requires the integration of various ways of knowing to approach feasible solutions and integrate perspectives of policy, politics, science and practice [26]. For some, a bio-inspired sustainable future requires a shifted worldview in which the underlying assumptions of an anthropocentric eco-industrial society are challenged and replaced by bioinclusive ethics that guide an ecological civilization [27].

## 6. Epistemological Influences in Transdisciplinary Fields of Study

In many applications (particularly those related to sustainability), BII is uniquely positioned as an interdisciplinary and transdisciplinary method of inquiry [23]. A transdisciplinary approach is common within BII, particularly as it is used to address the “wicked problems” of sustainability [28] and in the context of socioecological systems [12]. Although innovations resulting from BII are not always intended for more sustainably-oriented technologies [29], the “Biomimetic Promise” that innovation outcomes using a BII approach will be inherently more sustainable and effective is a widely promoted and accepted position [2]. BII innovators frequently position the application of biological metaphor to human problem solving as a natural solution set, creating a naturalistic fallacy that assumes what is natural is inherently good and sustainable without a wider consideration of the implications of innovation [3,30], or of the processes that underlie the generation of solutions in the natural world.

This is clearly exemplified in the field of computer science where applied genetic algorithms demonstrate the modern bioinspiration movement. Genetic algorithms are mathematical optimization algorithms that solve engineering and other problems by representing the unknown variables as strings of digits that mimic DNA. This biomimetic approach has been hugely successful producing innovations in a range of fields (e.g., ophthalmology [31], oncology [32], engineering [33], and economics [34]); however, such work is rarely framed with any reference to the natural world or sustainability. Since 2010, there have been over 338,000 articles and books mentioning the approach, yet only 6700 (1.9%) mentioning sustainability and even fewer (0.8%) mentioning the word nature (results from Google Scholar, https://scholar.google.com/) ignoring citations and patents. Search dated from 2010 until 13 February 2018).

This utilitarian bioinspired language can be contrasted to the position of BII experts such as Benyus who promotes biomimicry as innovation inspired by nature: “In a society accustomed to dominating or ‘improving’ nature, this respectful imitation is a radically new approach, a revolution really. Unlike the Industrial Revolution, the Biomimicry Revolution introduces an era based not on what we can extract from nature, but on what we can learn from her” and “The more our world functions like the natural world, the more likely we are to endure on this home that is ours, but not ours alone” ([1], p. 2). Benyus’ position evokes a more biophilic perspective towards the emulation of nature than those applying genetic algorithms. While both Benyus and the users of genetic algorithms are viewing the same biological phenomenon derived from reductionist methodologies, their interpretation of these models in biomimetic applications are a result of the social constructions they have created upon understanding the phenomenon. Benyus’ construction is the possibility of a sustainability utopia based on natural models, while the users of genetic algorithms circumvent the potential for a naturalistic fallacy and are merely translating a mathematical model without such value-laden assumptions. In this way, the scientific method of understanding demonstrates a performative idiom, in that it produces content that is not simply knowledge, but rather a material interpretation of observed phenomena [35].

In another example, such intellectual wars over evolutionary theory sparked a multi-decadal clash between leading theorists Gould and Dawkins and eventually led to two distinctly different underlying assumptions diverging from each other within various fields of biological research [36]. Gould espoused a view of evolution at the species level with a key role being given to environmental events [37,38,39,40]. By contrast, Dawkins has focused on genes as the units of selection [41,42], both during the early stages of life on earth and in more complex organisms—which he views as alliances of genes. There is an important lesson here for the future of BII, which is central to this paper: Gould and Dawkins presented their arguments not only in academic literature, but also in the public domain (largely via a series of popular science books). The distinctly different underlying assumptions behind these two ways of thinking about evolution have driven the research and interpretation of biological phenomena for generations of researchers. The impact of these epistemological assumptions should not be underestimated, as they have a profound influence on research agendas, innovation trajectories, and the human experience of natural systems.

We also argue in this paper that discussion can lead not just to disagreement, but also to resolution across disciplinary divides. Biologists examining organismal fields such as wildlife and botany may be more inclined to think holistically in systems and therefore these fields may lend more naturally to sustainability concepts, whereas molecular and cellular biologists may be more intellectually inclined to examine aspects of a system to extract benefits for society and therefore less intellectually trained to view systems with a sustainability lens.

## 7. A Proposed Path for Navigating the Tower of Babel in BII

BII scholars and practitioners could benefit from examining these disciplinary divides and assessing how the various BII fields can draw together under the banner of sustainability to grow a more useful epistemology. To aid more effective communication between these varying disciplines and epistemologies, we propose the following course of action. First, we recommend the overt identification of these epistemological variations in interdisciplinary research processes to bring this lack of alignment to the forefront. Second, we recommend careful consideration of what sustainability means across disciplines and how various team members relate to and evaluate the concept. Finally, further research and discourse is necessary to identify best practices in effective communication between specific disciplines and perhaps novel epistemological perspectives that can be applied in this unique BII context. Without adopting these recommendations, we suggest the possible positive societal and ecological impacts of BII are unlikely to develop to their full potential.

**Figure 2:**
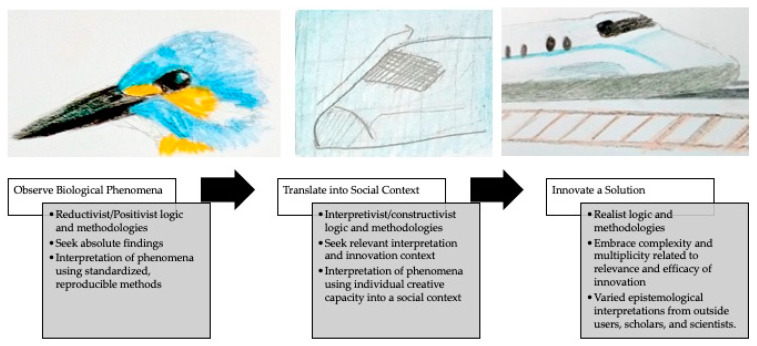
The epistemological shift of bioinspired innovation (BII).

The first recommendation suggests that interdisciplinary teams would benefit from a discussion of epistemological positioning and basic assumptions related to expectations of success in transdisciplinary processes. This area has been studied and theorized in other disciplinary contexts (e.g., [43,44,45,46], providing existing models to base these discussions and analyses around. Team members likely have numerous assumptions about individualized versions of success in BII. Some with more purist expectations may be aiming to copy the biological model as close to the natural phenomena as possible, perhaps relying on quantitative measures of performance success. Others may be aiming for the greatest benefit in solving a socioecological challenge and relying on qualitative indicators of success. The value of each perspective is readily debatable and while it may be challenging to establish consensus, an open discussion of these varied perceptions is likely to advance team alignment throughout a project, as is common practice across other academic communities and communities of practice. Figure 2 adds the commonly related epistemological positions to the phases of BII, which may facilitate deepened discussion across disciplines.

**Figure 3:**
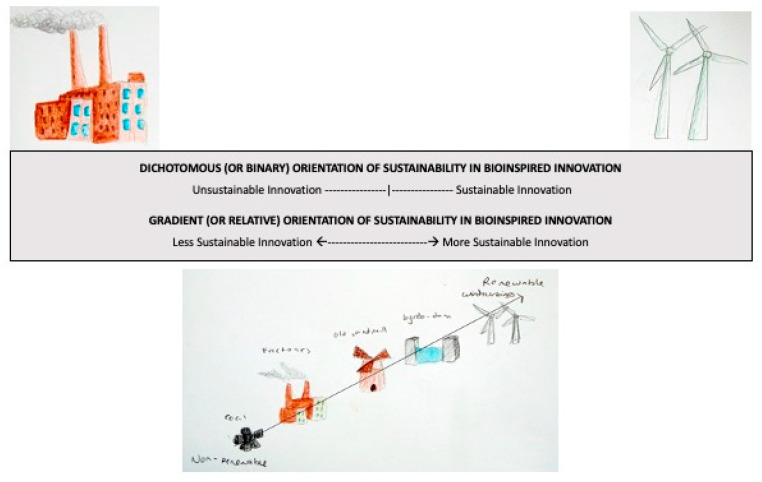
Dichotomous versus gradient orientation of sustainability in BII (adapted from [50], p. 226).

The second recommendation is that an exploration of views and perspectives related to sustainability is a necessary discussion for an interdisciplinary team to avoid misaligned expectations and intentions. Our position argues that sustainability intention should be viewed on a gradient, instead of a dichotomy of sustainable versus unsustainable to avoid the naturalistic fallacy in practical applications. Rather than viewing biomimicry as being a practice intended for sustainability, compared to biomimetics that is not (as suggested by [47,48,49] and others), it should be viewed on a spectrum of “more sustainable” to “less sustainable” dependent upon a number of factors including time scale of consideration and context of application. Figure 3 (adapted from a more general description of sustainability by McElroy, Jorna, and Engelen [50]) demonstrates the difference between a dichotomous (or binary) orientation of sustainability versus a gradient (or relative) orientation. The gradient orientation creates conceptual space to consider the myriad of possible perspectives amongst varied disciplines. In contrast, the dichotomous orientation may limit dialogue related to sustainability to a set of specific criteria rather than a systemic perspective, as has been advocated by numerous scholars (e.g., [51,52,53,54]).

**Figure 4:**
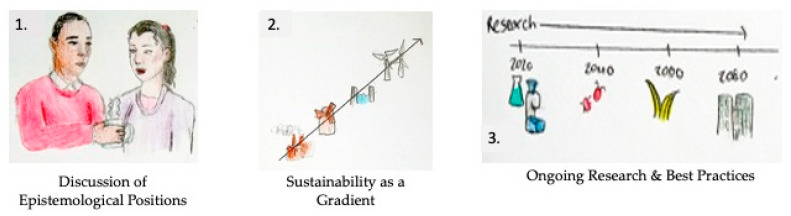
Three recommendations to improve the clarity of the dialogue amongst teams: (1) articulation of epistemological perspectives amongst team members; (2) application of a gradient orientation and (3) dialogue and further research.

The third recommendation is for continued research and establishment of best practices amongst multidisciplinary and transdisciplinary teams to develop novel methodological and epistemological perspectives that can contribute to the socially and ecologically relevant emulation of natural systems in innovation settings. This could be accomplished by the application of analysis strategies such as stakeholder engagement, needs assessment, and disciplinary tools related to socioecological performance unique to each team member’s area of expertise (e.g., life cycle analysis, cost-benefit analysis, environmental impact statements, etc.). While these types of considerations may not be appropriate for all BII-based teams, it is likely that innovation outcomes would benefit from more frequent and deepened consideration of epistemology and sustainability. The models presented in this paper could be used as foundational components of ongoing research to test the underlying assumptions of team processes and further explore how individuals experience and interpret bioinspired processes. Future research may include comparative case study analysis among and between teams with and without the aforementioned considerations. These recommendations are demonstrated in Figure 4.

## 8. Conclusions

This discussion of epistemological perspectives in BII has introduced the concept of an embedded epistemological shift. Throughout each step of this epistemological shift, BII innovators are unknowingly and inconsistently applying epistemological interpretations to their design and research processes. A lack of recognition of this shift from one epistemological perspective to the next and inconsistencies in defining sustainability amongst disciplines has material consequences for socioecological systems. However, BII innovators are largely unaware of this transition from objective observations that resulted from reductionist methods to value-laden implications embodied in novel technological applications. Furthermore, each of these steps has methodologically unique characteristics in comparison to the other steps, making the design process a complex transdisciplinary activity. This is especially evident in academic literature where BII research is scattered across discipline-specific journals, from engineering to sociology, via biology and computer science, making a thorough transdisciplinary perspective particularly challenging. Here, the authors have made three recommendations to reduce the potential ambiguities of the epistemological shift: (1) the deliberate articulation of epistemological perspectives amongst team members; (2) the application of a gradient orientation towards sustainability instead of a dichotomous orientation and (3) ongoing dialogue and further research to develop novel epistemological approaches to BII. Further consideration of the epistemological shift and its consequences will enable greater impact of BII, especially as it relates to sustainability.

## Appendix A. Glossary of Terms

Human Agency—The ability of an individual to act in a particular context or situation.

Epistemology—The study of knowing and the creation of knowledge.

Ethics—An aspect of moral philosophy that addresses questions of right and wrong, good and evil, morality, and justice.

Fallacy—An argument or position that is invalid because of faulty logic or reasoning.

Constructivism—The epistemological position that a phenomenon can only be understood in the context of meaning that is co-created with other people and within its environment.

Idiom—An expression used in common language in which the words used are not related to the meaning of the expression. E.g., ‘bite off more than you can chew.’

Interpretivist—An epistemological position that a phenomenon cannot be understood as an objective reality, but rather as a plurality of perceptions made by individual observers.

Naturalistic Fallacy—A logical fallacy that because something is natural or comes from nature, it is inherently “good” or “pure”.

Normativity—The societal designation as some actions or situations as good, desirable, appropriate, or permissible, while others are bad, undesirable, inappropriate, or impermissible.

Ontology—A philosophical study concerned with the nature of being and the existence of a phenomenon.

Performative – Language which acts as a kind of social action, such as a judge’s verdict, a promise, or a wedding ceremony.

Positivism—An epistemological position that a phenomenon can be understood as an external, singular, objective reality regardless of the observer’s perspective.

Sustainability—From the Brundland Report (1987): Development that “meets the needs of the present without compromising the ability of future generations to meet their own needs.” This definition has recently been expanded to include the Sustainable Development Goals as described by United Nations.

Utilitarian—Design which is intended to be useful, functional, or practical, rather than attractive, interesting, or provocative.
